# Reagent Tracker Dyes Permit Quality Control for Verifying Plating Accuracy in ELISPOT Tests

**DOI:** 10.3390/cells7010003

**Published:** 2018-01-03

**Authors:** Alexander Lehmann, Zoltan Megyesi, Anna Przybyla, Paul V. Lehmann

**Affiliations:** 1 Research and Development Department, CTL, Shaker Heights, OH 44122, USA; alexander.lehmann@immunospot.com (A.L.); zoltan.megyesi@immunospot.com (Z.M.); anna.przybyla@immunospot.com (A.P.); 2 Department of Cancer Immunology, Chair of Medical Biotechnology, Poznan University of Medical Sciences, 61-701 Poznan, Poland

**Keywords:** ImmunoSpot^®^, antigen screening, determinant mapping, CD4 cells, CD8 cells, T cells, RT dyes, audit trails for ELISPOT, regulated ELISPOT

## Abstract

ELISPOT assays enable the detection of the frequency of antigen-specific T cells in the blood by measuring the secretion of cytokines, or combinations of cytokines, in response to antigenic challenges of a defined population of PBMC. As such, these assays are suited to establish the magnitude and quality of T cell immunity in infectious, allergic, autoimmune and transplant settings, as well as for measurements of anti-tumor immunity. The simplicity, robustness, cost-effectiveness and scalability of ELISPOT renders it suitable for regulated immune monitoring. In response to the regulatory requirements of clinical and pre-clinical immune monitoring trials, tamper-proof audit trails have been introduced to all steps of ELISPOT analysis: from capturing the raw images of assay wells and counting of spots, to all subsequent quality control steps involved in count verification. A major shortcoming of ELISPOT and other related cellular assays is presently the lack of audit trails for the wet laboratory part of the assay, in particular, the assurance that no pipetting errors have occurred during the plating of antigens and cells. Here, we introduce a dye-based reagent tracking platform that fills this gap, thereby increasing the transparency and documentation of ELISPOT test results.

## 1. Introduction

ELISPOT assays are uniquely suited to detect individual antigen-specific T cells in peripheral blood mononuclear cells (PBMC) [1]. A strong-point of the ELISPOT assay is its sensitivity, which allows the visualization of even one antigen-specific T cell within one million PBMC [2]. In ELISPOT assays, PBMC are plated into 96- or 384-well PVDF membrane microtiter plates, which have been coated with cytokine-specific capture antibodies. Depending on the expected frequency of antigen-specific T cells in the PBMC being assayed, anywhere between 10^5^ and 10^6^ PBMC can be plated per well into 96-well plates (and between 3 × 10^4^ and 3 × 10^5^ PBMC per well into 384-well plates), resulting in a linear relationship between the number of PBMC plated and the antigen-specific T cells detected [3]. This is due to the fact that, in this PBMC concentration range, the cells form a monolayer on the bottom of the well [3], providing ideal conditions for T cell–APC (antigen presenting cell) interactions. The addition of antigen to select assay wells will result in the antigen being presented via APCs, and subsequent activation of the antigen-specific T cells. 

Upon activation, the antigen-specific T cells engage in the secretion of lineage-specific cytokines: Th1 cells will secrete IFN-γ, Th-2 cells IL-4 or IL-5, Th-17 cells IL-17 [4] and polyfunctional T cells will co-express cytokines such as IFN-γ, IL-2, and TNF-α [5]. The cytokine-specific capture antibodies present on the membrane will capture their specific analyte, resulting in a cytokine “spot” around the secreting T cell. These membrane-bound spots are then visualized by adding cytokine-specific detection antibodies, which can be done singly or in combinations of up to 4-plex [6]. Counting these spots (also called “spot forming units”, SFU), allows the enumeration of the antigen-specific T cells of each cytokine lineage within all PBMC plated per well. The direct contact between the secreting T cell and the detection surface results in the unique sensitivity of ELISPOT assays: the analyte is captured before it diffuses into the supernatant or is captured from the supernatant by high-affinity receptors present on bystander PBMC. In this way, even a single antigen-specific T cell can be reliably detected within one million PBMC, which is one hundred times more sensitive than flow cytometry with intracytoplasmic cytokine staining (ICS): for ICS, the detection limit is approximately 0.01% cells, that is, one in ten thousand cells [7].

Additionally, ELISPOT has been widely adopted due to its unique suitability for high-throughput testing. Hundreds of individual peptides can be tested on a single PBMC donor in large-scale epitope determinant mapping experiments [8,9,10,11]. The high throughput suitability of ELISPOT is enabled by a number of factors. First, there is the economy of cell utilization, which is of particular importance when it comes to clinical specimens, as PBMC from patients are only available in limited quantities. In ELISPOT assays, every cell plated is being interrogated. Pushing ELISPOT’s present limits, up to 30 antigens can be tested in 384-well format, using only one million PBMC [3]. The relatively simple workflow of ELISPOT also favors high throughput testing; essentially it is a two-step process in which just the PBMC and the antigen need to be plated into each pre-coated well. Last but not least, the streamlined and automated ELISPOT counting process enables high throughput testing. Although individual SFU can differ vastly in their diameter reflecting on the amount of analyte secreted by the individual T cells [12], as a population, the SFU generated by the repertoire of antigen-specific T cells will invariably follow a Log normal size distribution [13]. Knowing the rules for SFU size variations permits the use of automated gating and counting parameters for ELISPOT analysis [14]. Once the wet laboratory part of the ELISPOT assay itself has been concluded, the evaluation of 96-well plates, including scanning, analysis, and audit trail generation can progress at a rate of two minutes per plate, with the ability to analyze multiple plates with an automated plate loading attachment. 

Presently, the only major limitation to validated high throughput ELISPOT testing is the lack of audit trails for the plating of antigen and PBMC. When robotics are used for pipetting antigens and PBMC, one can assume that the experimental plan will be precisely implemented. However, most ELISPOT assays are currently pipetted by hand; leaving open the potential for pipetting mistakes. The slightest distraction or fatigue can lead to the experimenter losing track of how far (s)he has progressed with pipetting a particular antigen, and perhaps adding the wrong antigen to an assay well or group of wells. We have developed, and report here, a visual reagent tracking strategy to overcome this final limitation for validated high throughput ELISPOT testing.

## 2. Materials and Methods

### 2.1. PBMC Donors

PBMC were selected, based on their pre-established antigen reactivity, from the ePBMC library (CTL, Shaker Heights, OH, USA, www.immunospot.com/ImmunoSpot-ePBMC). The PBMC were thawed according to a protocol that has been optimized for the recovery of viable and functional cells [15]. The cells were plated within 2 h of thawing, as we found that “overnight resting” does not provide benefits towards the functionality of cryopreserved PBMC [16]. Additionally, it was reported that overnight resting changes the cytokine signatures of antigen-specific T cells [17]. More than 95% viable cells were recovered from all PBMC samples. The PBMC were adjusted to 3 × 10^6^ PBMC/mL in CTL-test medium (CTLT-005, from CTL), 100 μL of which (3 × 10^5^ cells) were plated per well into the ELISPOT assay.

### 2.2. Antigens

CEF-7 is CMV pp65(495–503), an HLA-A2-restricted immune dominant peptide of CMV that activates CD8 cells [18]. CEF-7 was from CTL (cat. #: CEF32-07-005) and was tested at a final concentration of 1 μg/mL. CMVgr2 antigen is CMV virion, UV inactivated, from Microbix (Mississauga, ON, Canada, cat. #: EL-01-02-001). CMVgr2 antigen requires processing and results in antigen presentation to CD4 cells [19]. It was tested at a final concentration of 30 μg/mL. Influenza A gr2 antigen—UV inactivated flu virus was from Microbix (cat. #: EL-13-02-001) and was used at 12.5 μg/mL. Mumps- and Measles gr.2 antigens (from Microbix, cat. #: EL-06-02-001 and cat. #: EL-04-02) were used at final concentrations 10 μg/mL and 50 μg/mL, respectively. P3H3 antigen (from Microbix, cat. #: EL-16-03-001), representing inactivated Epstein Bar virus (EBV), was tested at 30 µg/mL.

### 2.3. Reagent Tracker Dyes

A panel of 12 visually distinguishable food dyes were selected and assigned serial numbers A-L. These dyes, purchased as liquids, were diluted in sterile CTL Test Medium at a ratio of one to two. This stock solution was passed through a 0.2 μm filter for sterilization and stored at 4 °C. The concentration of dye present in each experiment is specified. The final dyes selected, C, D, E, and L, are now commercially available from CTL as CTL reagent tracker (RT) dyes 1, 2, 3 and 4, respectively (cat. #: CTL-RT1-010, CTL-RT2-010, CTL-RT3-010, CTL-RT4-010). 

### 2.4. Human Cytokine ELISPOT Assays

IFN-γ ELISPOT assays were done using ImmunoSpot^®^ kits from CTL (cat. #: CTL-HIFNGp-1/5M). The test procedure was performed according to the manufacturer’s recommendation. In brief, into IFN-γ capture-antibody pre-coated plates, the specified antigens were plated according to the plate layout described for each experiment in the Results section. The antigens were plated in a final volume of 100 μL per well dissolved in CTL Test Medium (CTLT-005) at the concentrations specified above. Antigen solutions were tested with or without admixing a tracker dye with the dye concentration specified. Medium alone—or medium supplemented with tracker dye at the specified concentration—constituted the negative control wells. 

The plates with antigen—and dye-stained antigen—were stored at 37 °C in a CO_2_ incubator all until the cells were plated, typically within an hour. PBMC were added at 3 × 10^5^ cells/well in 100 μL with wide-bore pipette tips. Plates were tapped on each side to make sure the PBMC distributed evenly across the well surface. The PBMC were cultured for 24 h with the antigen—or antigen/dye solution—at 37 °C and 9% CO_2_ in a humidified incubator. Thereafter the cells were discarded from the assay plate, detection antibody was added, and the plate-bound detection antibody was visualized by enzyme-catalyzed substrate precipitation. Prior to analysis, the plates were air-dried. 

ELISPOT plates were counted with an ImmunoSpot^®^ S6 Ultimate Reader by CTL. SFU were calculated automatically by the ImmunoSpot^®^ Software (Version 7, CTL, Shaker Heights, OH, USA) using the Autogate^TM^ function [14]. ELISPOT assays measuring human IL-1β, IL-6, IL-10, IL-12 and TNF-α were performed following the same protocol, using the corresponding ImmunoSpot^®^ kits by CTL. 

### 2.5. Statistical Analysis

Because ELISPOT counts follow Gaussian (normal) distribution among replicate wells [13], the Student’s *t*-test was used for identifying significant differences among test conditions, i.e., whether a dye was neutral, stimulatory or inhibitory. A *p*-value < 0.01 was considered as the cut off for a significant difference.

## 3. Results

### 3.1. Screening Candidate Reagent Tracker Dyes for Interference with T Cell Activation

**Figure 1:**
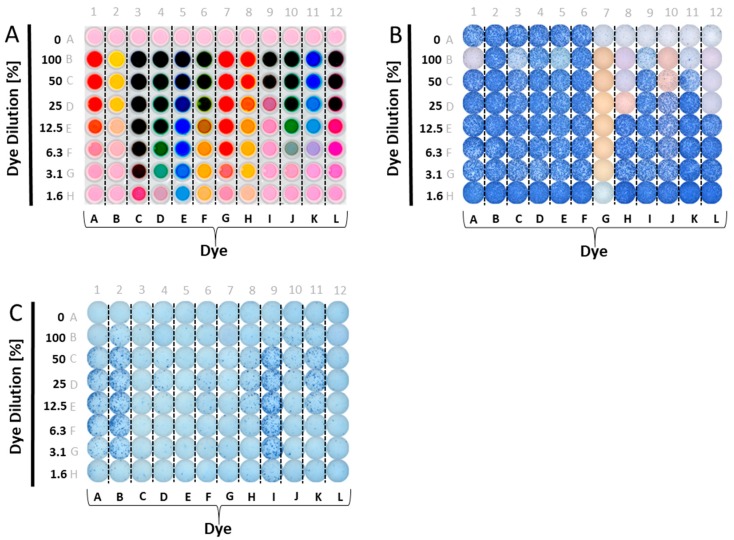
Testing candidate reagent tracker dyes for their interference with ELISPOT test results. (**A**) Candidate reagent tracker dyes were admixed to phenol red-containing test medium in the concentrations specified on the *Y* axis—a different dye for each column, as specified on the *X*-axis. Row A contains CEF-7 (wells A1-A6) or test medium alone (wells A7-A12). Each well contained 200 μL. A top-lit photograph of the experimental plate is shown. (**B**) The scanned well image of an ELISPOT plate is shown in which CEF 7 peptide-induced IFN-γ production by a single PBMC donor was measured. In row A, the PBMC were tested without the dyes present; wells A1–A6 are replicates containing the CEF-7 peptide at 1 μg/mL, and wells A7–12 are replicates for the medium control, with PBMC in medium alone. In all other wells CEF-7 peptide was present at 1 μg/mL, along with the dyes at the concentrations specified in the layout shown in (**A**); (**C**) The scanned image of an ELISPOT plate is shown in which IFN-γ production by a single donor was measured in the absence of antigen added; the PBMC were exposed only to the specified dyes or medium. In row A, in 12 replicate wells, PBMC were tested in medium alone, without the dyes present. In all other wells, the specified dyes were admixed to medium in the concentrations specified in the dye layout shown in (**A**). Note the occasional differential membrane staining in B vs. C: the staining seen at high dye concentrations with ethanol pre-wetting (**B**) was not seen without pre-wetting (**C**).

A panel of food dyes with clearly visibly distinct color profiles was selected. These were dissolved in CTL-test medium at a ratio of one to two (dye: 2× Medium, this constitutes the 100% stock solution), and plated in serial dilution. The optical presentation of such a plate is shown in Figure 1A. We then tested whether admixing these dyes with antigen would affect T cell activation. CEF-7, an HLA-Class 1-restricted peptide of CMV virus that activates CD8 cells [18], was used for the initial screening experiments. Figure 1B shows the results of these tests. Wells in Row A contain no added dyes, and serve as the reference values for IFN-γ production in the presence of CEF-7 antigen (wells A1–A6), or medium alone (the negative control, wells A7–A12). In rows B–H, in addition to CEF-7 at the same concentration, 1 µg/mL, the dyes were also present in decreasing concentrations, as specified. As can be readily seen, several dyes were inhibitory/toxic at high concentrations. Dye G was fully inhibitory, even at 1.6%. 

### 3.2. Screening Candidate Reagent Tracker Dyes for Stimulatory Activity

Using a similar plate layout, we tested whether the dyes have a stimulatory effect on PBMC (Figure 1C). Row A was assigned as the medium control reference value (without dye added), with no wells of the plate containing antigen. Instead of admixing the dyes with antigen, as before, here the dyes were plated in serial dilution in medium alone. As can be seen, several dyes exerted a strong stimulatory effect, in particular, dyes A, B, and I. Moderate, but clearly significant, stimulation was seen for dyes F, H, J, and K. Only dyes C, E, L and D were not significantly stimulatory.

**Figure 2:**
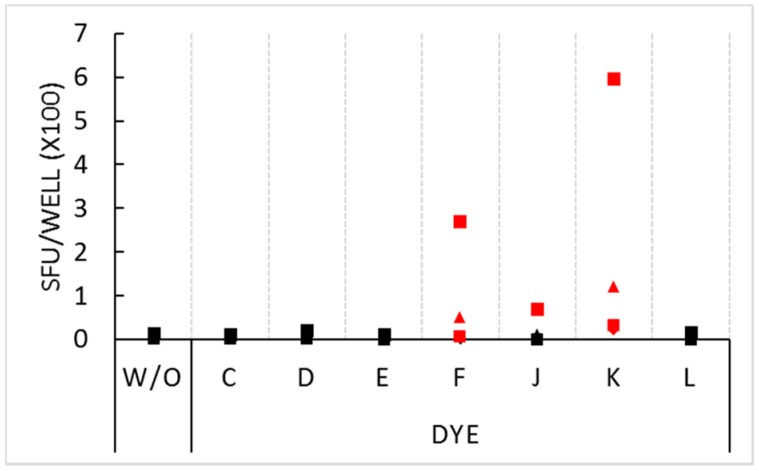
Stimulatory effect of select dyes at 1.5% concentration. PBMC of four donors, distinguished by four symbols, were plated at 3 × 10^5^ cells per well in medium alone (without inclusion of dyes or antigen, W/O), or with the dyes specified on the *X* axis (also without antigen added). Each condition was tested in three replicate wells with the mean spot count shown on the *Y* axis. SFU counts that exceeded the threshold for statistical significance (*p* < 0.01) by the Student’s *t*-test are represented with red symbols.

In the above experiment, PBMC from five donors were tested and generated confirmatory results. In repeat experiments, we tested for the non-inhibitory dyes’ stimulatory activity on four different PBMC donors. The stimulatory effect for dyes F and K was seen on these donors as well (Figure 2), however the extent of stimulation showed marked inter-donor differences ranging from very strong (>200 SFU per well) in some donors, to weaker, but significant, stimulation in the other donors. One of the four donors was stimulated by dye J: this donor’s PBMC generated 68 SFU in the presence of the dye compared to 13 SFU in medium alone.

As a side note to this paper that addresses the suitability of food dyes for tracking reagents, it was surprising that eight of 12 commonly used food dyes—admixed generously to foods we consume daily—were not found to be neutral, as we initially expected, but had profound immune stimulatory effects. It is a striking possibility, therefore, that such dyes serve as adjuvants, and contribute to the development of food allergies, either directed against the food dyes themselves, or to food antigens to which they are admixed, or both.

### 3.3. Four Finalist Dyes Are Neither Inhibitory nor Stimulatory over a Wide Concentration Range

**Figure 3:**
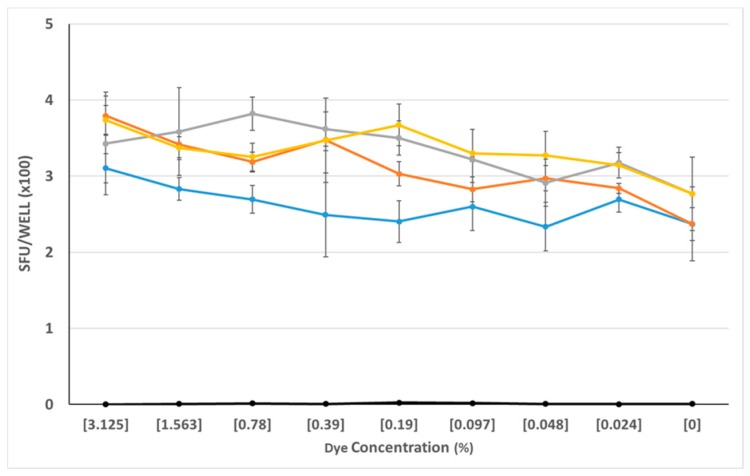
Neutral effect of the four finalist dyes over a wide concentration range. PBMC of a CEF-7 reactive donor were tested in an IFN-γ ELISPOT assay for CEF-7—induced production of the cytokine vs. spot formation in the medium control. The test was done in the absence of added dye (0% dye concentration), or in the presence of the specified concentrations of dyes C, D, E, and L, distinguished by colors. (The grey line is dye C, dye E is in yellow, dye D is orange, and dye L is represented by the blue line.) SFU counts in all medium control wells—in the presence or absence of dye—were less than 10/well and are seen as the black symbols overlaying the *X* axis. The data shown have been obtained from a single donor, and is representative of three additional donors tested.

The above screening experiments ruled out eight of 12 dyes as reagent tracker candidates. Only four dyes, C, D, E, and L proved so far to be neither significantly stimulatory nor inhibitory. As with the other dyes, we saw dose-dependent effects. We tested the selected dyes at concentrations that were still clearly visibly discernable, and from the data obtained from previous experiments, appeared to be biologically neutral. We re-tested these four dyes (C, D, E, and L) over a wide concentration, ranging from 3% to 0.024%, and using PBMC from 4 additional donors. As can be seen in Figure 3, these four dyes behaved neutrally over the entire dye concentration range tested: neither the antigen-triggered T cell response to CMVgr2, nor spot formation in the medium control was affected. 

### 3.4. Four Finalist Dyes Behave Neutrally for 17 Donors

Due to the considerable inter-donor variability seen above for some dyes (Figure 2), we next tested the four selected dyes on a wide array of PBMC donors. Based on the dose-response studies described above (Figure 3), and visual discernibility of the dye(s), we determined the optimal concentrations of 0.39%, 0.39%, 0.09% and 1.56% for dyes C, D, E, and L, respectively. At these concentrations, the four dyes were admixed to CMVgr2 antigen (which recalls CMV-specific CD4 cells [19]), and tested 17 PBMC donors that previously have been shown to display various frequencies of CD4 memory cells specific for this antigen. The test was done comparing the CMVgr2-induced SFU without dye admixed, with SFU induced in the presence of CMVgr2 plus dye, with each condition tested in nine replicate wells. 

**Figure 4:**
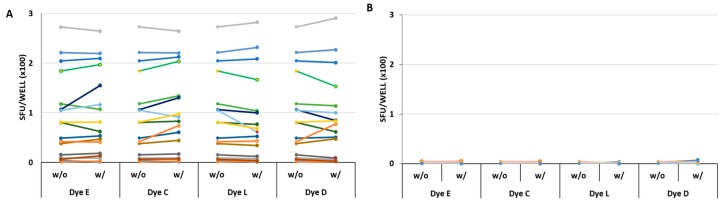
Verifying the four finalist dyes’ lack of inhibitory or stimulatory effect. (**A**) The CMVgr2 antigen response in PBMC from 17 donors. The PBMC of 17 donors are distinguished by colors. The PBMC were tested in the absence (w/o) or in the presence (w/) of the specified dyes present at 0.39%, 0.39%, 0.09% and 1.56% for dyes C, D, E, and L, respectively. The corresponding SFU counts, representing means of triplicate wells, are connected by a line. None of these data pairs reached statistically significant difference: the *p*-values for comparing the donor groups tested with or without dyes were 0.89, 0.82, 0.87 and 0.96 for dyes E, C, L and D respectively; (**B**) The medium control results for all 17 donors in the presence or absence of the four dyes. No statistically significant differences were seen: the *p*-values for comparing the donor groups tested with or without dyes were 0.43, 0.22, 0.05 and 0.07 for dyes E, C, L and D respectively.

As seen in Figure 4, the SFU counts were similar in all 17 donors in the presence or the absence of the four selected dyes. Statistical comparison of SFU counts under the two conditions revealed no significant differences for the CMVgr2 antigen-triggered responses (Figure 4A). In the same experiment, using the same 17 PBMC donors, the background SFU formation was also tested using medium alone, or medium with the four dyes admixed individually (Figure 4B) No significant stimulatory effect was seen for any of the dyes. 

We extended this testing for CD4 cell recall responses to EBV(P3H3), Measles, Mumps, and Influenza. As above, PBMC donors that had pre-established SFU counts for each of these antigens, were selected from the ePBMC database, and the cells were tested with and without admixed dyes. The paired SFU counts for EBV(P3H3), Measles, Mumps, and Influenza are shown in Supplementary Materials Figures S1–S4, respectively. Statistical comparison of the duplicate wells for each test case, with or without dye, did not show a significant difference, neither for the comparison antigen alone vs. antigen plus dye, nor medium alone vs. medium plus dye (Figure 4B). Similar results were obtained testing 9 additional PBMC donors for CEF-7 peptide-induced response (Supplementary Materials Figure S5). 

As some of the dyes stimulated vigorous IFN-γ production in the absence of antigen (see above), we also tested whether the four selected dyes would activate cells of the innate immune system. PBMC of 8 donors were plated into ELISPOT assays measuring IL-6, TNF-α, IL-1β, IL-10, and IL-12 and cultured with RT dyes C, D, E, and L at the above noted concentrations for 24 h. None of these cytokines were induced by any of the four dyes, as compared to control wells with PBMC cultured in media alone (see Supplementary Materials Figures S6–S10). Thus, it was determined that none of the selected dyes stimulated cells of the innate immune system at the concentrations used for reagent tracking. 

### 3.5. Four Finalist Dyes Allow Visual Audit Trails for Antigen Plating

**Figure 5:**
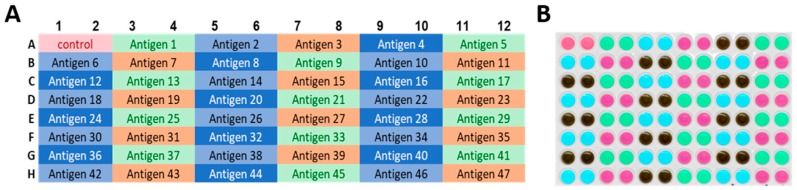
Visual quality control for plating of antigen. (**A**) The plate layout for an experiment is shown in which 47 antigens are to be tested on a plate, each in duplicate wells. The four selected reagent tracker dyes have been assigned to the antigens in a revolving order, as shown by the four background colors assigned to the data fields. Wells A1 and A2 are the negative controls, with cells in phenol red containing medium only, without antigen/RT dye added; (**B**) A back-lit photograph of the ELISPOT plate is shown after the RT-dye-labelled antigens have been plated. The finalist dye concentrations were used, 0.39%, 0.39%, 0.09% and 1.56% for dyes C, D, E, and L, respectively in phenol red free medium.

The above data show that from 12 food dyes, only four are suited to be admixed with antigen for reagent tracking. These four selected reagent tracker dyes at the optimized concentration (CTL RT dyes), provide visual guidance for plating antigen, as well as audit trails for documenting the accuracy of antigen plating. Figure 5A shows the plate layout for a high throughput ELISPOT assay in which 165 antigens (peptides) are to be tested. The four RT dyes have been assigned in a revolving sequence to the antigens: the first two wells, A1 and A2, constitute the medium control (i.e., no antigen, no dye). The next two wells, A3 and A4, contain antigen 1 with RT dye 1. In the following 2 wells, A5 and A6, antigen 2 is to be plated with RT dye 2 antigen 3 is to follow in wells A7 and A8 with RT dye 3, and then antigen 4 in wells A9 and A10 with RT dye 4. Here the dye sequence starts over again: antigen 5 with RT dye 1 in wells A11 and A10, antigen 6 with RT dye 2 in wells B1 and B2, and so on until antigen 47 on this plate. The sequence continues on the next plate with antigen 48 through antigen 164 (not shown for Plate 2). While pipetting the RT dye-labelled antigens, the color scheme of the plate-layout guides the experimenter: Figure 5B shows a photograph of the actual assay plate after the RT dye-labeled antigens have been plated. Such a plate photograph is suited as an audit trail: the regular pattern of coloration matching the plate layout verifies that, indeed, the experimental plan has been precisely implemented, i.e., all antigens have been plated, in duplicate, into the wells they have been assigned. 

### 3.6. Four Finalist Dyes Allow Automated Electronic Audit Trails for Antigen Plating

A photograph of the plate containing the RT dyes can serve as an audit trail, from the regulatory standpoint. However, it is preferable to be able to generate an automated electronic audit record. For that to be feasible, the four RT dyes need to be clearly distinguishable from each other by image analysis, which they are (AL, manuscript in preparation). Ideal auditing would not only verify whether and which RT dye (antigen) has been plated, but whether the PBMC had been added as well, completing the assay. Addition of the PBMC results in 50% RT dye dilution: as 100 μL per well of PBMC are to be added to the 100 μL of RT dye-labelled antigen). Therefore, it is possible to generate fully automated electronic audit trails that not only distinguish the individual RT dyes but also, whether PBMC have been added. To enable such electronic audit trail generation, we have implemented a dedicated ImmunoSpot^®^ RT-Software Suite. Its experimental validation has been completed and is the subject of a manuscript in preparation. 

## 4. Conclusions

Here, we demonstrate the feasibility of using RT dyes to assist the implementation of ELISPOT experiments. RT dyes provide visual guidance in the error-prone process of manual plating of antigens and PBMC. Subsequently, photographs of the finalized plates can provide an audit trail of the experiment’s implementation to verify that no qualitative or quantitative pipetting error has occurred. This approach should not only assist regulated studies but also help to implement complex experiments in which a multitude of antigens are to be tested. ELISPOT is already one of the few cellular test systems suited for regulated work. The RT dye approach constitutes a major step towards introducing further regulatory transparency. In addition to T cell ELISPOT, any bioassay that involves testing a multitude of reagents can benefit from a similar RT approach. However, for each of these additional test systems the neutrality of the RT dyes needs to be established. 

## Supplementary Materials

Supplementary materials are available on line www.mdpi.com/2073-4409/7/1/3/s1. Figure S1: Verifying the four finalist dyes’ lack of inhibitory or stimulatory effect on P3H3 Antigen response in PBMC from 9 donors. Figure S2: Verifying the four finalist dyes’ lack of inhibitory or stimulatory effect on Measles Antigen response in PBMC from 9 donors. Figure S3: Verifying the four finalist dyes’ lack of inhibitory or stimulatory effect on Mumps Antigen response in PBMC from 9 donors. Figure S4: Verifying the four finalist dyes’ lack of inhibitory or stimulatory effect on Influenza Antigen response in PBMC from 9 donors. Figure S5: Verifying the four finalist dyes’ lack of inhibitory or stimulatory effect on HCMV pp65(495–503) peptide response in PBMC from 9 donors. Figure S6: Verifying the four finalist dyes’ lack of inhibitory or stimulatory effect on cells of the innate immune system: the IL-6 data. Figure S7: Verifying the four finalist dyes’ lack of inhibitory or stimulatory effect on cells of the innate immune system: the TNF-α data. Figure S8: Verifying the four finalist dyes’ lack of inhibitory or stimulatory effect on cells of the innate immune system: the IL-1β data. Figure S9: Verifying the four finalist dyes’ lack of inhibitory or stimulatory effect on cells of the innate immune system: the IL-10 data. Figure S10: Verifying the four finalist dyes’ lack of inhibitory or stimulatory effect on cells of the innate immune system: the IL-12 data.
