# Supplementary material for: Reagent Tracker Dyes Permit Quality Control for Verifying Plating Accuracy in ELISPOT Tests
Source: Cells. 2018 Jan 3;7(1):3. doi: 10.3390/cells7010003 (PMC5789276; doi:10.3390/cells7010003)
Supplement: Supplementary file 1 [file cells-07-00003-s001.pdf]

Article

Reagent Tracker Dyes permit quality control for verifying plating accuracy in ELISPOT tests

Alexander Lehmann[1], Zoltan Megyesi[1], Anna Przybyla [1, 2], and Paul V. Lehmann [1,*]

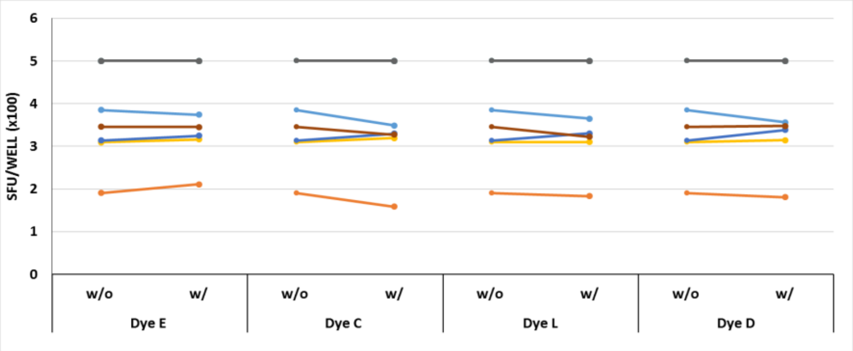

Suppl. Figure 1. Verifying the four finalist dyes' lack of inhibitory or stimulatory effect on P3H3 Antigen response in PBMC from 9 donors. Except for the antigen used, Legend to Figure 4 applies. The p values for comparing the donor groups tested with or without dyes were 0.96, 0.90, 0.95 and 0.99 for dyes E, C, L and D respectively. The medium control data for these donors are shown in Fig, 4B.

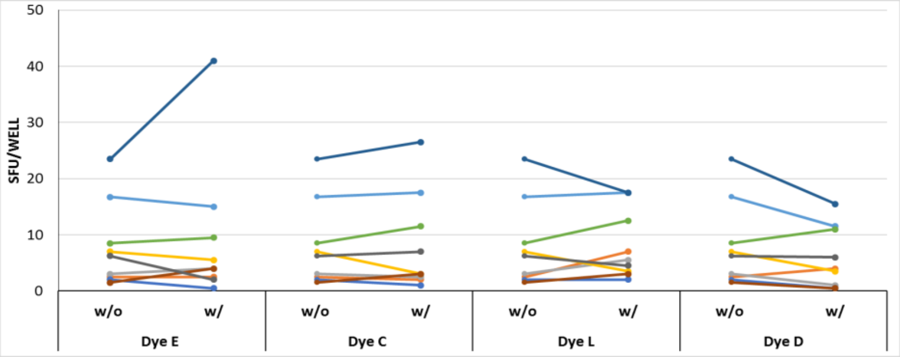

Suppl. Figure 2. Verifying the four finalist dyes' lack of inhibitory or stimulatory effect on Measles Antigen response in PBMC from 9 donors. Except for the antigen used, Legend to Figure 4 applies. Note that in this Figure the SFU counts are relatively low (< 40 per well) compared to the other supplemental figures, where they are in the hundreds. Due to the Normal distribution of SFU counts, relative differences in low numbers can appear striking to the eye, whereas –like for all data pairs shown here – they do not reach statistical significance. The p values for comparing the donor groups tested with or without dyes were 0.65, 0.84, 0.95 and 0.54 for dyes E, C, L and D respectively. The medium control data for these donors are shown in Fig, 4B.

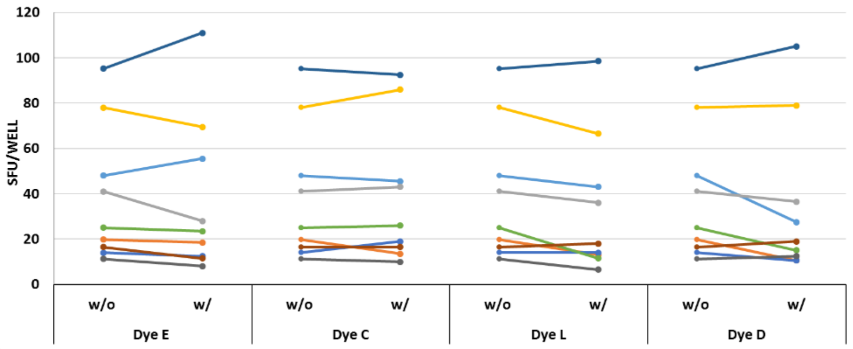

Suppl. Figure 3. Verifying the four finalist dyes' lack of inhibitory or stimulatory effect on Mumps Antigen response in PBMC from 9 donors.   Except for the antigen used, Legend to Figure 4 applies. The p values for comparing the donor groups tested with or without dyes were 0.92, 0.91, 0.75 and 0.81 for dyes E, C, L and D respectively. The medium control data for these donors are shown in Fig, 4B.

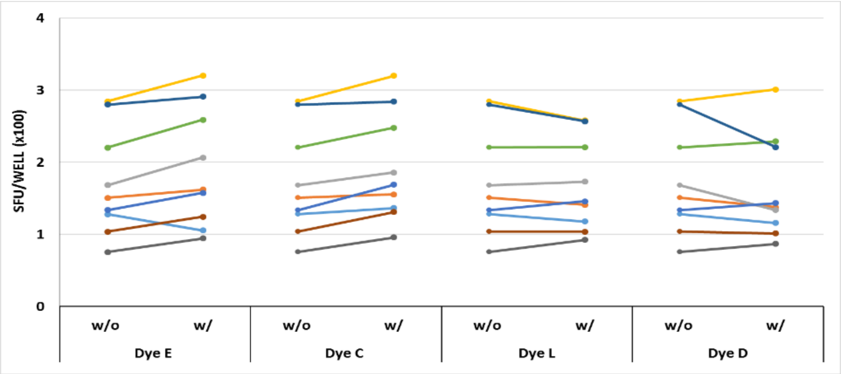

Suppl. Figure 4. Verifying the four finalist dyes' lack of inhibitory or stimulatory effect on Influenza Antigen response in PBMC from 9 donors.   Except for the antigen used, Legend to Figure 4 applies. The p values for comparing the donor groups tested with or without dyes were 0.56, 0.50, 0.89 and 0.81 for dyes E, C, L and D respectively. The medium control data for these donors are shown in Fig, 4B.

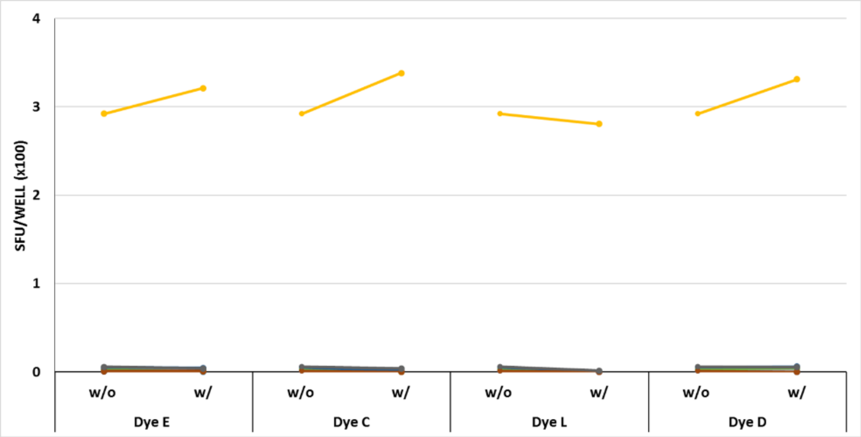

Suppl. Figure 5. Verifying the four finalist dyes' lack of inhibitory or stimulatory effect on HCMV pp65(495–503) peptide response in PBMC from 9 donors.   Except for the antigen used, Legend to Figure 4 applies. The p values for comparing the donor groups tested with or without dyes were 0.96, 0.91, 0.95 and 0.93 for dyes E, C, L and D respectively. The medium control data for these donors are shown in Fig, 4B.

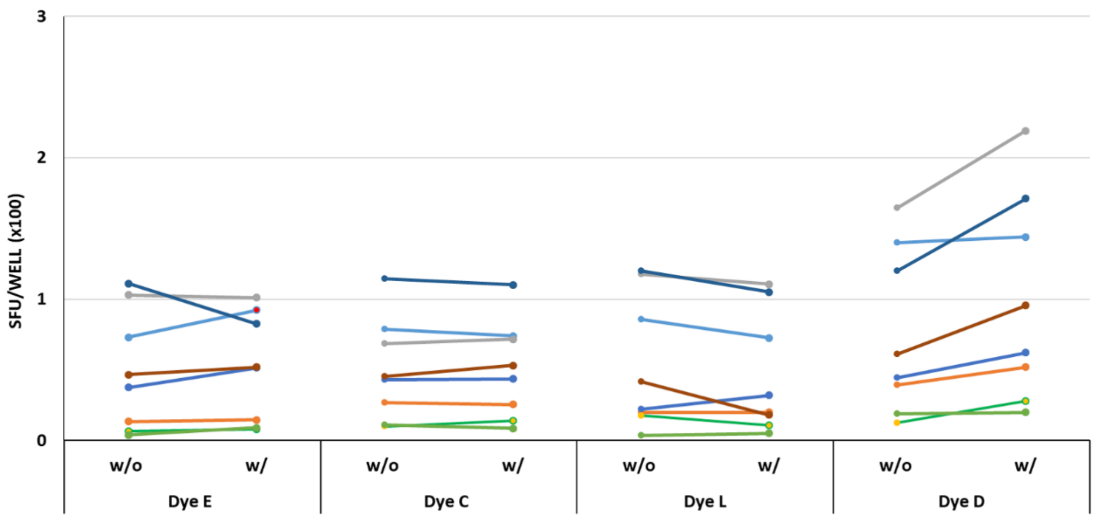

Suppl. Fig 6. Verifying the four finalist dyes' lack of inhibitory or stimulatory effect on cells of the innate immune system: the IL-6 data. PBMC of 8 donors are distinguished by color. The PBMC were tested in medium alone, in the absence (w/o) or in the presence (w/) of the specified dyes present at 0.09%, 0.39%, 1.56% and 0.39% for Dyes E, C, L, and D, respectively. ELISPOT assays detecting cells secreting IL-6 were performed. The corresponding SFU counts, representing means of triplicate wells, are connected by a line. None of these data pairs reached statistically significant difference. The p values for comparing the donor groups tested with or without dyes were 0.71, 0.87, 0.12 and 0.013 for dyes E, C, L and D respectively.

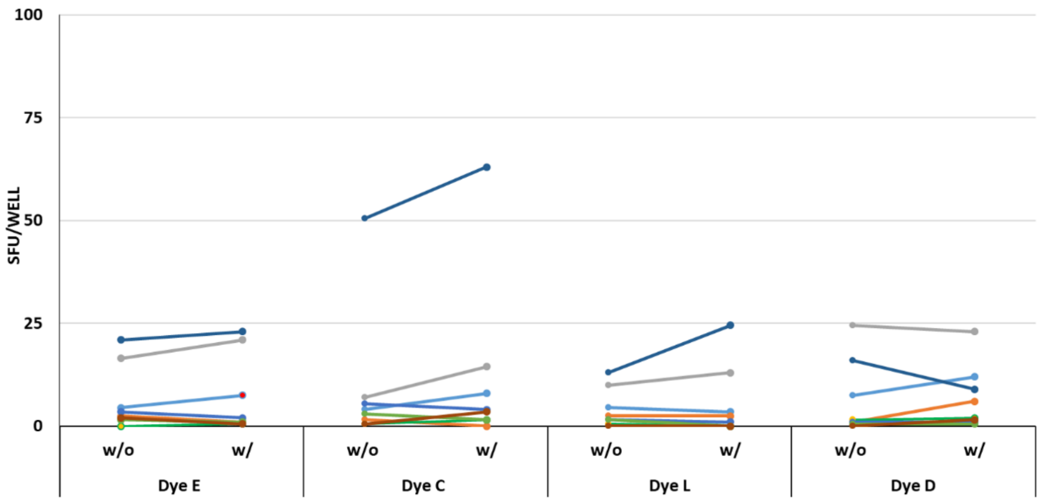

Suppl. Fig 7. Verifying the four finalist dyes' lack of inhibitory or stimulatory effect on cells of the innate immune system: the TNF-α data. PBMC of 8 donors are distinguished by color. The PBMC were tested in medium alone, in the absence (w/o) or in the presence (w/) of the specified dyes present at 0.09%, 0.39%, 1.56% and 0.39% for Dyes E, C, L, and D, respectively. ELISPOT assays detecting cells secreting TNF-α were performed. The corresponding SFU counts, representing means of triplicate wells, are connected by a line. None of these data pairs reached statistically significant difference. The p values for comparing the donor groups tested with or without dyes were 0.47, 0.14, 0.40 and 0.78 for dyes E, C, L and D respectively.

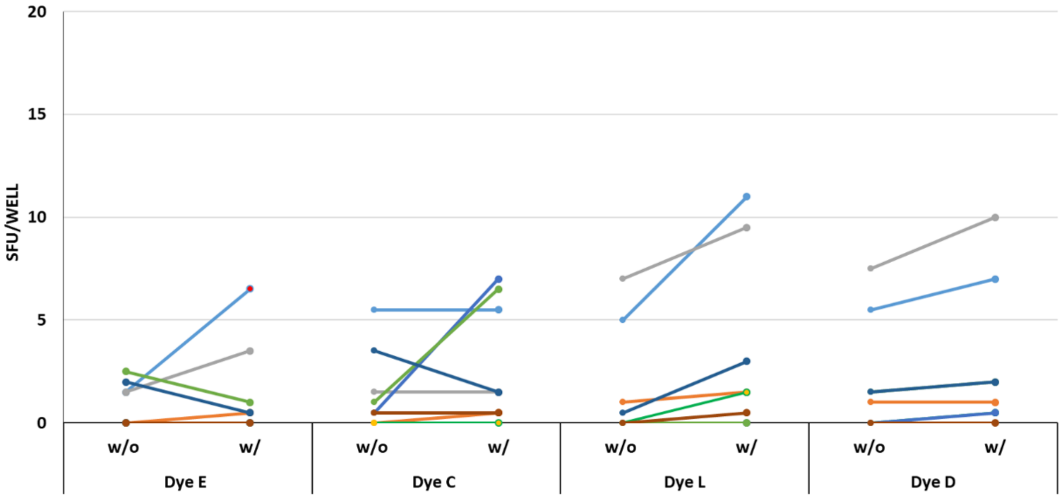

Suppl. Fig 8. Verifying the four finalist dyes' lack of inhibitory or stimulatory effect on cells of the innate immune system: the IL-1β data. PBMC of 8 donors are distinguished by color. The PBMC were tested in medium alone, in the absence (w/o) or in the presence (w/) of the specified dyes present at 0.09%, 0.39%, 1.56% and 0.39% for Dyes E, C, L, and D, respectively. ELISPOT assays detecting cells secreting IL-1β were performed. The corresponding SFU counts, representing means of triplicate wells, are connected by a line. None of these data pairs reached statistically significant difference. The p values for comparing the donor groups tested with or without dyes were 0.48, 0.26, 0.05 and 0.04 for dyes E, C, L and D respectively.

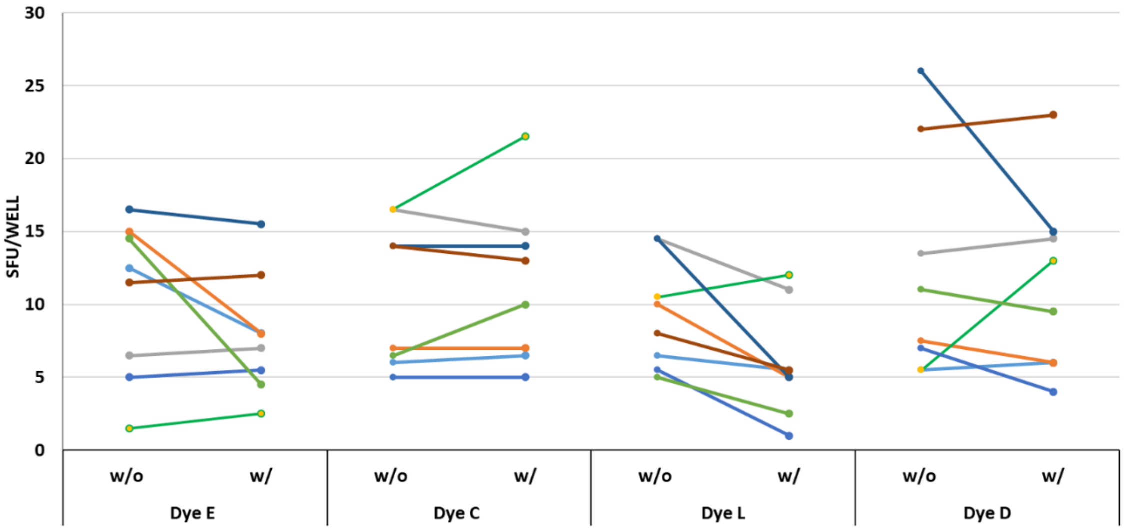

Suppl. Fig 9. Verifying the four finalist dyes' lack of inhibitory or stimulatory effect on cells of the innate immune system: the IL-10 data. PBMC of 8 donors are distinguished by color. The PBMC were tested in medium alone, in the absence (w/o) or in the presence (w/) of the specified dyes present at 0.09%, 0.39%, 1.56% and 0.39% for Dyes E, C, L, and D, respectively. ELISPOT assays detecting cells secreting IL-10 were performed. The corresponding SFU counts, representing means of triplicate wells, are connected by a line. None of these data pairs reached statistically significant difference. The p values for comparing the donor groups tested with or without dyes were 0.13, 0.34, 0.02 and 0.65 for dyes E, C, L and D respectively.

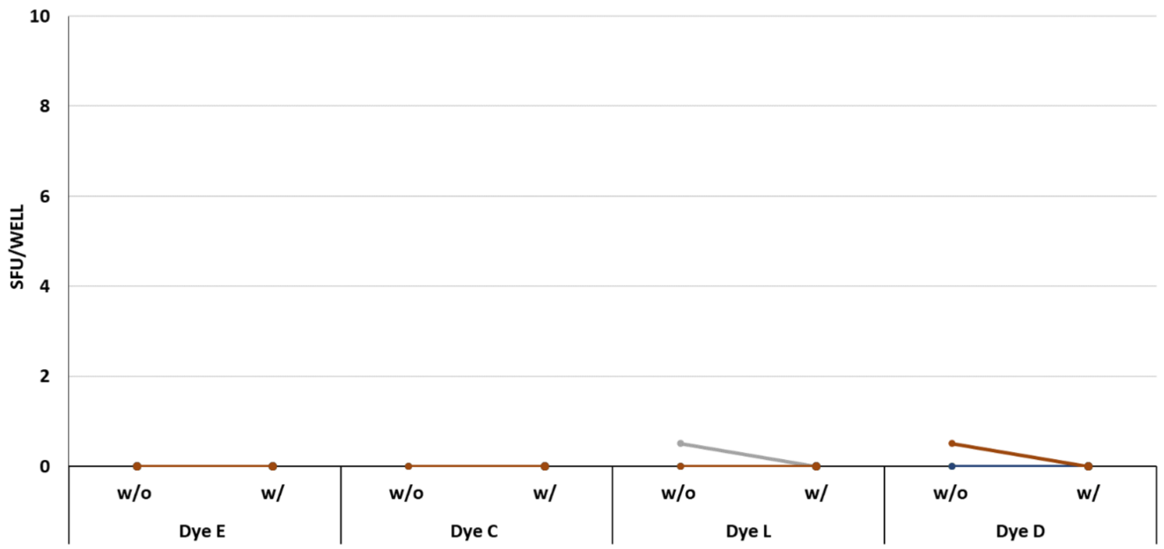

Suppl. Fig 10. Verifying the four finalist dyes' lack of inhibitory or stimulatory effect on cells of the innate immune system: the IL-12 data. PBMC of 8 donors are distinguished by color. The PBMC were tested in medium alone, in the absence (w/o) or in the presence (w/) of the specified dyes present at 0.09%, 0.39%, 1.56% and 0.39% for Dyes E, C, L, and D, respectively. ELISPOT assays detecting cells secreting IL-12 were performed. The corresponding SFU counts, representing means of triplicate wells, are connected by a line. None of these data pairs reached statistically significant difference. The p values for comparing the donor groups tested with or without dyes were <0,99, <0.99, and 0.35 for dyes E, C, L and D respectively.
